# Supplementary material for: RNA-Seq Analysis of Extradomain A and Extradomain B Fibronectin as Extracellular Matrix Markers for Cancer
Source: Cells. 2023 Feb 21;12(5):685. doi: 10.3390/cells12050685 (PMC10000746; doi:10.3390/cells12050685)
Supplement: Supplementary file 1 [file cells-12-00685-s001.zip › cells-2162539-supplementary.pdf]

Supplementary Table S1

Sample Count by Cohort																
Cancer Type	Normal Tissues			Primary Tumors												
	Total Count	*TCGA*	*GTEx*	Total Count	Pathologic T				Pathologic N		Pathologic M		Pathologic Stage			
					T1	T2	T3	T4	N-	N+	M0	M1	I	II	III	IV
**ACC**	128	*0*	*128*	77	8	38	8	17	62	9	0	0	9	36	15	15
**BLCA**	28	*19*	*9*	407	3	119	191	58	235	128	195	11	2	130	140	133
**BRCA**	292	*113*	*179*	1091	278	621	134	40	506	550	894	22	182	617	248	20
**CESC**	13	*3*	*10*	304	145	101	20	9	130	60	115	10	0	0	0	0
**CHOL**	9	*9*	*0*	36	19	12	5	0	26	5	28	5	19	9	1	7
**COAD**	349	*41*	*308*	286	6	44	195	40	165	120	192	40	44	110	82	40
**DLBC**	0	*0*	*0*	47	0	0	0	0	0	0	0	0	0	0	0	0
**ESCA**	666	*13*	*653*	181	31	41	84	4	73	87	130	9	18	76	55	9
**GBM**	1152	*0*	*1152*	152	0	0	0	0	0	0	0	0	0	0	0	0
**HNSC**	99	*44*	*55*	518	47	135	98	174	174	243	184	1	27	70	80	266
**KICH**	53	*25*	*28*	66	19	22	16	2	34	5	29	2	21	25	14	6
**KIRC**	100	*72*	*28*	530	247	61	160	10	213	12	380	67	266	57	123	81
**KIRP**	60	*32*	*28*	288	181	33	59	2	48	27	91	9	170	21	52	15
**LGG**	1152	*0*	*1152*	508	0	0	0	0	0	0	0	0	0	0	0	0
**LIHC**	160	*50*	*110*	369	178	93	80	13	249	4	263	4	169	86	85	5
**LUAD**	347	*59*	*288*	513	166	276	46	18	326	171	342	25	274	122	83	26
**LUSC**	338	*50*	*288*	498	111	289	70	22	312	174	404	7	242	161	84	7
**MESO**	0	*0*	*0*	87	14	24	31	12	42	37	54	3	10	16	45	16
**OV**	88	*0*	*88*	419	0	0	0	0	0	0	0	0	0	0	0	0
**PAAD**	171	*4*	*167*	178	5	23	134	3	45	118	76	4	21	147	3	4
**PCPG**	281	*3*	*278*	177	0	0	0	0	0	0	0	0	0	0	0	0
**PRAD**	152	*52*	*100*	495	0	0	0	0	321	75	0	0	0	0	0	0
**READ**	10	*10*	*0*	91	4	13	63	10	38	50	63	12	12	24	33	13
**SARC**	2	*2*	*0*	258	0	0	0	0	0	0	0	0	0	0	0	0
**SKCM**	813	*1*	*812*	102	1	5	10	84	58	28	97	3	2	66	26	3
**STAD**	210	*36*	*174*	414	21	86	179	113	121	268	360	27	58	121	169	41
**TGCT**	0	*0*	*0*	132	72	50	5	0	45	13	111	4	53	12	14	0
**THCA**	338	*59*	*279*	504	122	143	160	22	203	201	252	9	283	52	112	55
**THYM**	2	*2*	*0*	119	0	0	0	0	0	0	0	0	0	0	0	0
**UCEC**	91	*13*	*78*	180	0	0	0	0	0	0	0	0	0	0	0	0
**UCS**	78	*0*	*78*	57	0	0	0	0	0	0	0	0	0	0	0	0
**UVM**	0	*0*	*0*	79	0	0	0	0	51	0	50	4	0	0	0	0
**Totals**	**7182**	**712**	**6470**	**9163**	**1678**	**2229**	**1748**	**653**	**3477**	**2385**	**4310**	**278**	**1882**	**1958**	**1464**	**762**

Supplementary Table S1. Sample Sizes. Table of TCGA diseases and corresponding sample sizes for normal tissues and primary tumors. Normal tissue sample sizes are broken down by TCGA and GTEx samples. Primary tumors are broken down by cohort, including by various pathologic staging methods.

Supplementary Figure S1

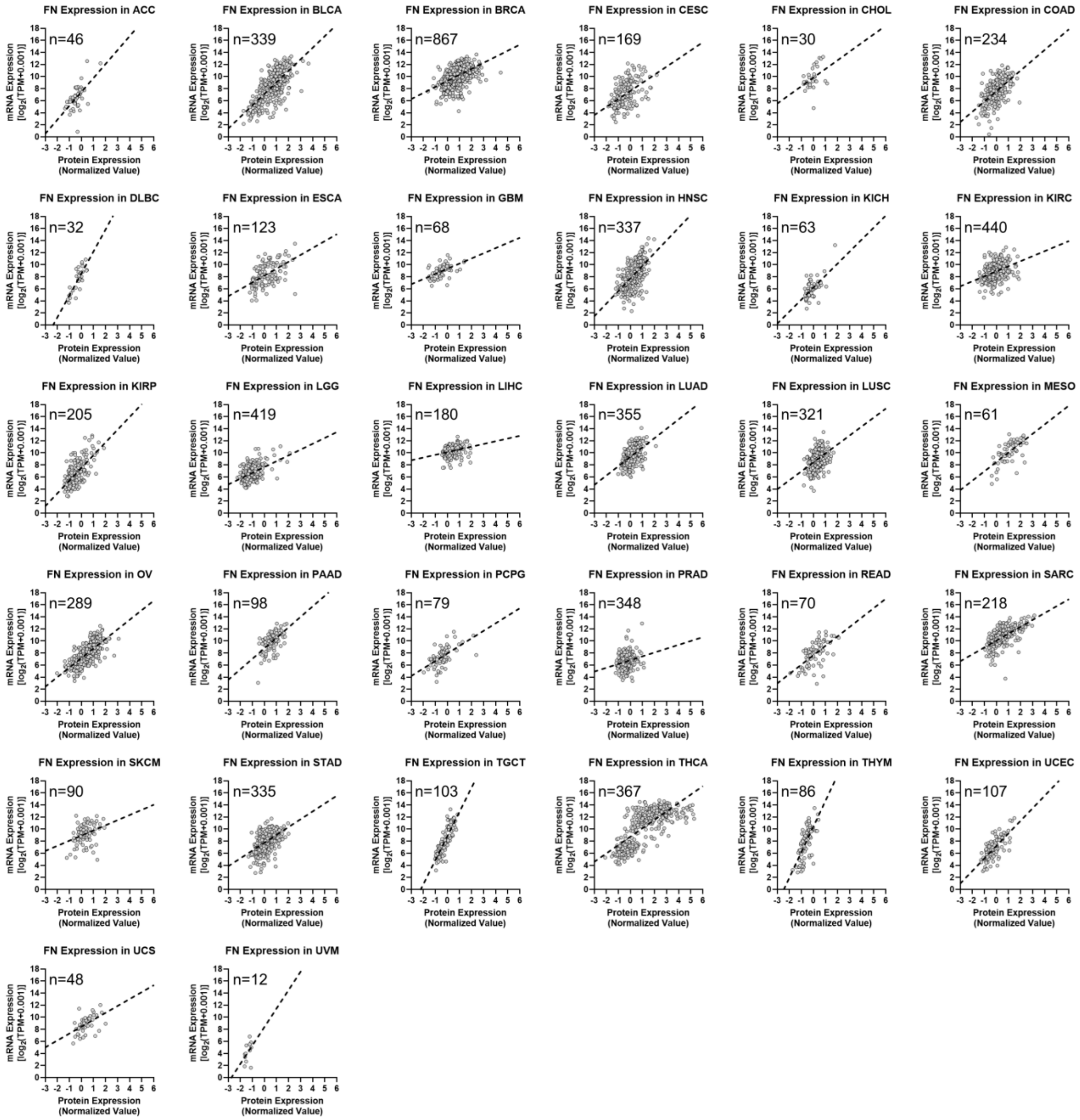

Supplementary Figure S1. Fibronectin mRNA expression (*FN1* gene) versus fibronectin protein expression separated by cancer type. There is an overall positive trend of increasing mRNA expression with increasing protein expression in every cancer type. Sample sizes for each cancer type are indicated in each respective figure. Sample sizes shown here differ from the total primary tumor counts for each cancer type in **Supp. Table 1** due to sample exclusion caused by missing protein expression data, thus reducing the sample size for this analysis.

Supplementary Table S2

Ensembl			RefSeq		Alternative Splicing			Protein	
Name	Transcript ID	cDNA	Transcript ID	cDNA	EDA	EDB	IIICS	AA's	Exons
-	-	-	XM_017003692	8132	Y	Y	V0	2357	46
-	-	-	XM_005246402	8324	Y	Y	V64	2421	47
FN1-201	ENST00000323926	8708	NM_001306129	8297	Y	Y	V89	2446	47
-	-	-	XM_024452769	8417	Y	Y	V95	2452	46
FN1-203	ENST00000354785	8390	NM_212482	8390	Y	Y	V120	2477	46
FN1-209	ENST00000432072	7759	NM_001306130	7760	-	Y	V0	2267	45
-	-	-	NM_001365522	7952	-	Y	V64	2331	46
-	-	-	NM_001365521	8027	-	Y	V89	2356	46
-	-	-	NM_001365519	8045	-	Y	V95	2362	45
-	-	-	NM_001365517	8120	-	Y	V120	2387	45
-	-	-	NM_001365524	7757	Y	-	V0	2266	45
FN1-212	ENST00000446046	7952	NM_212478	7949	Y	-	V64	2330	46
FN1-202	ENST00000336916	8435	NM_002026	8024	Y	-	V89	2355	46
-	-	-	NM_001365520	8042	Y	-	V95	2361	45
FN1-206	ENST00000359671	8524	NM_001365518	8117	Y	-	V120	2386	45
FN1-205	ENST00000357867	7898	NM_212474	7487	-	-	V0	2176	44
FN1-207	ENST00000421182	8103	NM_001306132	7679	-	-	V64	2240	45
FN1-211	ENST00000443816	7762	NM_001306131	7754	-	-	V89	2265	45
-	-	-	NM_001365523	7772	-	-	V95	2271	44
FN1-204	ENST00000356005	7846	NM_212476	7847	-	-	V120	2296	44

Supplementary Table S2. List of all possible alternative splicing combinations of ECM fibronectin. All possible combinations of alternative splicing are shown, with the corresponding splicing characteristics, amino acid length of the final protein, and the number of exons in the mature mRNA. Where applicable, the corresponding transcript names, IDs, and cDNA lengths from the Ensembl and RefSeq libraries are provided with their corresponding alternative splicing characteristics. The 10 ECM fibronectins from the Ensembl library are used in this study.

Supplementary Table S3

Ensembl			RefSeq		Transcript Type	Alt. Splicing		Protein	
Name	Transcript ID	cDNA	Transcript ID	cDNA		EDA	EDB	AA's	Exons
FN1-208	ENST00000426059	2388	NM_054034	2388	coding	-	-	657	13
FN1-210	ENST00000438981	723	-	-	coding	-	-	241	4
FN1-213	ENST00000456923	3720	-	-	coding	-	Y	1103	21
FN1-214	ENST00000460217	804	-	-	non-coding	-	-	-	-
FN1-215	ENST00000461974	571	-	-	non-coding	-	-	-	-
FN1-216	ENST00000469569	629	-	-	non-coding	-	-	-	-
FN1-217	ENST00000471193	496	-	-	non-coding	-	-	-	-
FN1-218	ENST00000473614	568	-	-	non-coding	-	-	-	-
FN1-219	ENST00000474036	1058	-	-	non-coding	Y	-	-	-
FN1-220	ENST00000480024	576	-	-	non-coding	-	-	-	-
FN1-221	ENST00000480737	384	-	-	non-coding	-	-	-	-
FN1-222	ENST00000485567	584	-	-	non-coding	-	-	-	-
FN1-223	ENST00000490833	699	-	-	non-coding	Y	-	-	-
FN1-224	ENST00000492816	9171	-	-	non-coding	Y	Y	-	-
FN1-225	ENST00000494446	676	-	-	non-coding	-	-	-	-
FN1-226	ENST00000496542	695	-	-	non-coding	-	-	-	-
FN1-227	ENST00000498719	2433	-	-	non-coding	-	-	-	-

Supplementary Table S3. List of short fibronectin transcripts in the Ensembl library. Where applicable, the corresponding transcript names, IDs, and cDNA lengths from the Ensembl and RefSeq libraries are provided with the corresponding transcript type, alternative splicing characteristics, and protein structure. Just one short fibronectin transcript corresponds to a known fibronectin protein – ENST00000426059, migration stimulating factor.

Supplementary Table S4

FN1 and Alternative Splice Variant Expression Normalized to Normal Tissue Expression								
Cancer Type	Sample Size		*FN1* Gene		Exon A		Exon B	
	Normal	Tumor	Normal Tissue	Primary Tumor	Normal Tissue	Primary Tumor	Normal Tissue	Primary Tumor
ACC	128	77	1.0000 ± 0.6692	4.1435 ± 12.7642	1.0000 ± 0.9099	2.5222 ± 7.1233	1.0000 ± 3.6085	3.6085 ± 10.2473
BLCA	28	407	1.0000 ± 0.9600	1.3219 ± 2.1185	1.0000 ± 1.1458	0.7545 ± 1.4407	1.0000 ± 0.9419	0.9419 ± 1.5619
BRCA	292	1091	1.0000 ± 0.7664	8.4374 ± 7.3495	1.0000 ± 1.0620	14.2768 ± 14.2305	1.0000 ± 9.5761	9.5761 ± 8.9922
CESC	13	304	1.0000 ± 0.6784	1.0588 ± 1.9865	1.0000 ± 0.9248	1.1314 ± 2.5457	1.0000 ± 0.9501	0.9501 ± 1.8537
COAD	349	286	1.0000 ± 0.9417	0.6002 ± 0.7931	1.0000 ± 1.2363	0.9483 ± 1.4763	1.0000 ± 0.6558	0.6558 ± 0.8904
ESCA	666	181	1.0000 ± 0.9332	0.7995 ± 1.3604	1.0000 ± 1.1767	1.8846 ± 3.5028	1.0000 ± 0.9642	0.9642 ± 1.6178
GBM	1152	152	1.0000 ± 1.0945	18.4856 ± 18.2224	1.0000 ± 1.6374	19.9746 ± 31.1553	1.0000 ± 20.0425	20.0425 ± 23.0437
HNSC	99	518	1.0000 ± 1.9076	7.3923 ± 17.2120	1.0000 ± 2.2103	11.6908 ± 29.7691	1.0000 ± 7.7161	7.7161 ± 18.2281
KICH	53	66	1.0000 ± 0.8295	2.3026 ± 11.9676	1.0000 ± 1.1194	7.6372 ± 51.1221	1.0000 ± 3.4673	3.4673 ± 20.1228
KIRC	100	530	1.0000 ± 0.9919	4.8906 ± 5.7736	1.0000 ± 1.1196	4.7617 ± 5.2775	1.0000 ± 5.3044	5.3044 ± 5.7938
KIRP	60	288	1.0000 ± 0.7741	3.2160 ± 8.8394	1.0000 ± 0.9472	2.2709 ± 6.0691	1.0000 ± 3.5869	3.5869 ± 12.8417
LGG	1152	508	1.0000 ± 1.0945	3.7652 ± 7.2043	1.0000 ± 1.6374	7.0209 ± 21.7095	1.0000 ± 4.4846	4.4846 ± 8.9559
LIHC	160	369	1.0000 ± 0.4299	1.9749 ± 1.1086	1.0000 ± 0.9527	4.5765 ± 5.0475	1.0000 ± 2.6388	2.6388 ± 1.8166
LUAD	347	513	1.0000 ± 0.5074	0.7360 ± 0.8711	1.0000 ± 0.5595	1.0707 ± 1.5099	1.0000 ± 0.8177	0.8177 ± 0.9617
LUSC	338	498	1.0000 ± 0.5067	0.5458 ± 0.6799	1.0000 ± 0.5947	0.8174 ± 1.3884	1.0000 ± 0.6293	0.6293 ± 0.8254
OV	88	419	1.0000 ± 0.7705	2.0910 ± 2.8651	1.0000 ± 1.0042	2.6336 ± 4.4426	1.0000 ± 2.1045	2.1045 ± 3.2931
PAAD	171	178	1.0000 ± 4.6454	20.8991 ± 19.9512	1.0000 ± 4.8732	22.8846 ± 26.3040	1.0000 ± 23.9148	23.9148 ± 24.7221
PCPG	281	177	1.0000 ± 0.4116	0.4233 ± 0.6149	1.0000 ± 0.5507	0.5672 ± 0.9600	1.0000 ± 0.5343	0.5343 ± 0.8191
PRAD	152	495	1.0000 ± 1.4688	0.4121 ± 1.1527	1.0000 ± 1.8002	0.4493 ± 0.8338	1.0000 ± 0.5045	0.5045 ± 1.4441
READ	10	91	1.0000 ± 0.6667	1.8238 ± 2.2330	1.0000 ± 0.7795	1.5152 ± 1.9235	1.0000 ± 1.4718	1.4718 ± 1.8272
SKCM	813	102	1.0000 ± 1.6309	0.1931 ± 0.2097	1.0000 ± 1.6605	0.1555 ± 0.2041	1.0000 ± 0.2161	0.2161 ± 0.2598
STAD	210	414	1.0000 ± 1.7814	1.2729 ± 1.4017	1.0000 ± 1.8877	2.2715 ± 2.9304	1.0000 ± 1.4848	1.4848 ± 1.8098
THCA	338	504	1.0000 ± 1.9076	41.1509 ± 43.1256	1.0000 ± 1.6165	55.1068 ± 71.7984	1.0000 ± 52.7086	52.7086 ± 56.8121
UCEC	91	180	1.0000 ± 0.8025	0.6643 ± 1.4083	1.0000 ± 0.7241	0.5142 ± 1.2110	1.0000 ± 0.5454	0.5454 ± 1.2284
UCS	78	57	1.0000 ± 0.7987	2.0496 ± 2.1538	1.0000 ± 0.7196	1.7766 ± 2.3457	1.0000 ± 1.8877	1.8877 ± 2.2652
**Average**	**7169**	**8405**	**1.0000 ± 0.0000**	**5.2260 ± 9.1488**	**1.0000 ± 0.0000**	**6.7685 ± 11.8037**	**1.0000 ± 0.0000**	**6.0303 ± 11.3735**

Supplementary Table S4. Expression data for the *FN1* gene and all transcripts containing the EDA or EDB exons. Dataset produced from the TCGA and GTEx databases, showing sample sizes and average ± standard deviation of normalized expression of the *FN1* gene, Exon A, and Exon B for normal tissue and primary tumors for each cancer type included in the analysis. Cancer types that were excluded from analysis had fewer than 10 normal tissue or primary tumor samples.

Supplementary Table S5

ECM Fibronectin and Alternative Splice Variant Expression Normalized to Normal Tissue Expression								
Cancer Type	Sample Size		ECM Fibronectin		EDA-FN		EDB-FN	
	Normal	Tumor	Normal Tissue	Primary Tumor	Normal Tissue	Primary Tumor	Normal Tissue	Primary Tumor
ACC	128	77	1.0000 ± 0.8147	2.3333 ± 5.1108	1.0000 ± 0.9333	2.5833 ± 6.9951	1.0000 ± 0.9318	2.8831 ± 6.9693
BLCA	28	407	1.0000 ± 1.1941	0.7218 ± 1.3308	1.0000 ± 1.1742	0.7599 ± 1.4562	1.0000 ± 1.1432	0.4746 ± 0.8949
BRCA	292	1091	1.0000 ± 1.0899	11.7374 ± 12.8435	1.0000 ± 1.1325	15.5047 ± 15.5244	1.0000 ± 1.5024	14.3656 ± 16.7640
CESC	13	304	1.0000 ± 0.9209	1.1677 ± 2.3990	1.0000 ± 0.9552	1.2917 ± 2.8930	1.0000 ± 1.1654	0.9000 ± 2.4286
COAD	349	286	1.0000 ± 1.1926	1.3767 ± 2.2559	1.0000 ± 1.2711	1.0887 ± 1.7145	1.0000 ± 1.2772	0.7921 ± 1.5061
ESCA	666	181	1.0000 ± 1.2623	2.2472 ± 3.5895	1.0000 ± 1.2359	2.1532 ± 3.9807	1.0000 ± 1.3536	1.7872 ± 2.9124
GBM	1152	152	1.0000 ± 1.3310	15.1677 ± 18.5480	1.0000 ± 1.7119	22.3195 ± 32.5624	1.0000 ± 1.4539	22.3427 ± 50.5408
HNSC	99	518	1.0000 ± 2.2552	8.6302 ± 18.9887	1.0000 ± 2.2921	12.3831 ± 31.5085	1.0000 ± 2.5872	10.1559 ± 25.7445
KICH	53	66	1.0000 ± 1.1714	4.3365 ± 24.6129	1.0000 ± 1.1696	8.0166 ± 53.7062	1.0000 ± 1.2442	9.9580 ± 64.4335
KIRC	100	530	1.0000 ± 1.4342	3.4541 ± 3.7299	1.0000 ± 1.1393	4.7743 ± 5.2953	1.0000 ± 1.7664	4.1436 ± 4.5964
KIRP	60	288	1.0000 ± 1.0066	3.6525 ± 15.8933	1.0000 ± 0.9873	2.3328 ± 6.2361	1.0000 ± 1.2816	6.0674 ± 36.9811
LGG	1152	508	1.0000 ± 1.3310	6.3113 ± 15.2202	1.0000 ± 1.7119	7.8163 ± 24.8694	1.0000 ± 1.4539	5.6159 ± 11.4749
LIHC	160	369	1.0000 ± 1.1132	2.5818 ± 2.6808	1.0000 ± 1.1103	5.4047 ± 6.0215	1.0000 ± 1.3321	10.5842 ± 16.7214
LUAD	347	513	1.0000 ± 0.5711	1.2500 ± 1.8153	1.0000 ± 0.5612	1.2088 ± 1.7136	1.0000 ± 0.8531	2.4176 ± 4.3692
LUSC	338	498	1.0000 ± 0.6456	0.8832 ± 1.4846	1.0000 ± 0.6143	0.9277 ± 1.5723	1.0000 ± 0.8982	1.9575 ± 3.7337
OV	88	419	1.0000 ± 1.0297	3.1328 ± 5.6840	1.0000 ± 1.0832	3.0665 ± 5.1674	1.0000 ± 0.9066	1.3386 ± 3.2729
PAAD	171	178	1.0000 ± 5.3141	28.7942 ± 38.0742	1.0000 ± 5.1661	24.3167 ± 28.1375	1.0000 ± 5.4805	31.9525 ± 43.9665
PCPG	281	177	1.0000 ± 0.6017	0.8167 ± 1.3214	1.0000 ± 0.5999	0.7346 ± 1.2574	1.0000 ± 1.1687	4.5226 ± 7.3351
PRAD	152	495	1.0000 ± 2.0657	0.6674 ± 2.3211	1.0000 ± 1.9456	0.4932 ± 0.9183	1.0000 ± 1.7950	0.6747 ± 1.9892
READ	10	91	1.0000 ± 0.8029	1.4545 ± 1.9721	1.0000 ± 0.7779	1.5205 ± 1.9324	1.0000 ± 0.8052	0.9758 ± 1.3752
SKCM	813	102	1.0000 ± 1.6863	0.0843 ± 0.1049	1.0000 ± 1.6607	0.1541 ± 0.2040	1.0000 ± 1.6372	0.1182 ± 0.2809
STAD	210	414	1.0000 ± 1.8695	3.1111 ± 3.5595	1.0000 ± 1.9259	2.4912 ± 3.1450	1.0000 ± 1.9362	2.6072 ± 3.9671
THCA	338	504	1.0000 ± 1.4261	58.7022 ± 77.0177	1.0000 ± 1.6864	62.4100 ± 81.6540	1.0000 ± 1.6380	92.4559 ± 112.7638
UCEC	91	180	1.0000 ± 0.8766	0.5972 ± 1.4067	1.0000 ± 0.7757	0.5673 ± 1.3505	1.0000 ± 1.0321	0.4903 ± 1.1529
UCS	78	57	1.0000 ± 0.9151	1.7462 ± 2.6477	1.0000 ± 0.7725	1.9464 ± 2.6797	1.0000 ± 1.0667	2.3368 ± 2.8367
**Average**	**7169**	**8405**	**1.0000 ± 0.0000**	**6.5983 ± 12.5204**	**1.0000 ± 0.0000**	**7.4506 ± 13.2494**	**1.0000 ± 0.0000**	**9.2767 ± 18.9099**

Supplementary Table S5. Expression data for ECM fibronectin and all full length transcripts containing the EDA or EDB exons. Dataset produced from the TCGA and GTEx databases, showing sample sizes and average ± standard deviation of normalized expression of ECM fibronectin, EDA-FN, and EDB-FN for normal tissue and primary tumors for each cancer type included in the analysis. Cancer types that were excluded from analysis had fewer than 10 normal tissue or primary tumor samples.

Supplementary Figure S2

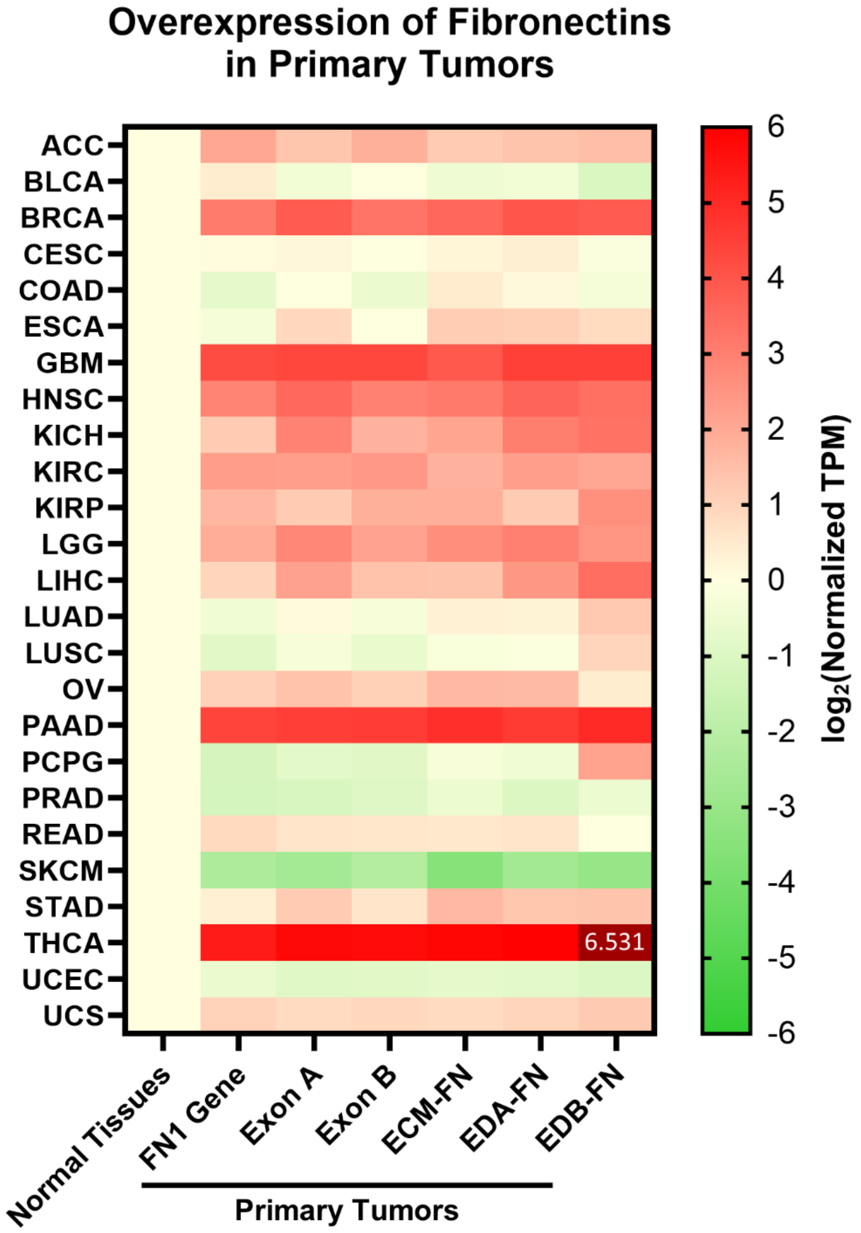

Supplementary Figure S2. Heat map detailing the overexpression of the *FN1 Gene* and all transcript groups presented in this study. TPM expression values in primary tumors for each expression group were normalized to corresponding normal tissues for each cancer type. As such, the normalized expression in normal tissue for every expression group is 1. To remove repetitive results, the normalized expression in normal tissues is shown just once. In addition, the scale bar was kept consistent with that in Figures 1 and 3. One overexpression value – EDB-FN in THCA – exceeds the maximum value of the scale bar, so the overexpression value is stated in the heat map.

Supplementary Figure S3

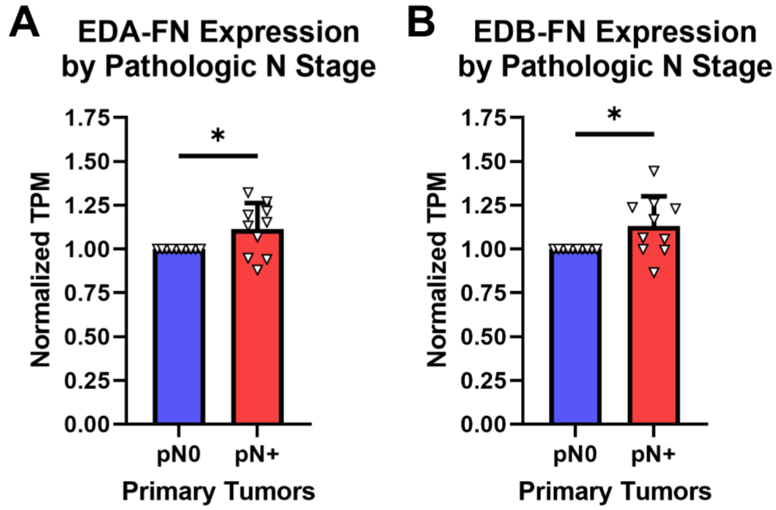

Supplementary Figure S3. EDA-FN and EDB-FN expression in other cancer types with pathologic N staging information. (A) EDA-FN and **(B)** EDB-FN expression in cancer types in node negative (N0) or node positive (N+) primary tumors, excluding head and neck squamous cell carcinoma. Cancer types were excluded from analysis if the sample size of either node negative or node positive patients was fewer than 10. Significance: *, $p<0.05$.

Supplementary Figure S4

A

Test (columns); Grount Truth (rows)

	Neg	Pos
**Neg**	TN	FP
**Pos**	FN	TP

B

Clinical N Stage (Clinical Test) (columns); Pathologic N Stage (Ground Truth) (rows)

	cN0	cN+	
**pN0**	139	28	**167**
**pN+**	58	175	**233**
	**197**	**203**	**400**

C

$$Prevalence = \frac{Total\ number\ of\ pathologic\ positives\ (TP + FN)}{Total\ number\ of\ patients\ (TP + TN + FP + FN)} = \frac{233}{400} = 0.583$$

$$Accuracy = \frac{True\ positives\ (TP) + True\ negatives\ (TN)}{Total\ number\ of\ patients\ (TP + TN + FP + FN)} = \frac{175 + 139}{400} = \frac{314}{400} = 0.785$$

$$Precision = \frac{True\ positives\ (TP)}{Total\ number\ of\ clinical\ positives\ (TP + FP)} = \frac{175}{203} = 0.862$$

$$Sensitivitity = \frac{True\ positives\ (TP)}{Total\ number\ of\ pathologic\ positives\ (TP + FN)} = \frac{175}{233} = 0.751$$

$$Specificity = \frac{True\ negatives\ (TN)}{Total\ number\ of\ pathologic\ negatives\ (TN + FP)} = \frac{139}{167} = 0.832$$

Supplementary Figure S4. Confusion matrix for binary classification and calculation of statistics. (A) Structure of a standard confusion matrix, showing the predictive test and the ground truth. Correct test outcomes are labeled true negative (TN) or true positive (TP), whereas incorrect test outcomes are labeled false negative (FN) or false positive (FP). **(B)** Structure of confusion matrix for head and neck squamous cell carcinoma (HNSC) lymph node diagnosis, with clinical N stage as the test and pathologic N stage as the ground truth. **(C)** Calculation of statistics from the confusion matrix from the data in (B).

Supplementary Figure S5

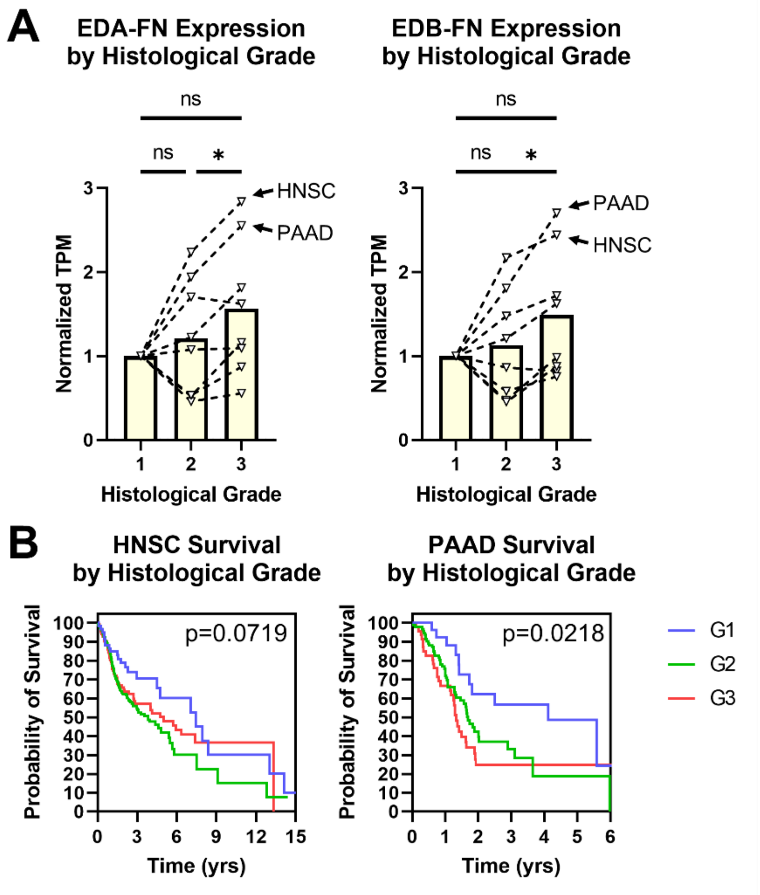

Supplementary Figure S5. Oncofetal fibronectin expression in other cancer types with histological grading information. (A) EDA-FN and EDB-FN expression in various cancer types by histological grade. Cancer types were excluded from analysis if the sample size in any cohort was fewer than 10. Grade 4 tumors were additionally excluded from analysis due to the small sample sizes in all but two cancer types. **(B)** Kaplan-Meier survival curves for HNSC and PAAD separated out by histological grade. HNSC and PAAD were selected because they most significantly overexpressed both EDA-FN and EDB-FN in high grade tumors.

Supplemental Table S6

Median Survival Times Based on High and Low Oncofetal Fibronectin Expression

Cancer Type	Median Survival (yrs)		% Change
	Low EDA-FN	High EDA-FN	
BLCA	5.40	1.95	-63.9%
BRCA	11.69	10.05	-14.0%
CHOL	3.84	3.34	-13.0%
ESCA	3.46	1.87	-46.1%
GBM	1.38	0.99	-28.3%
HNSC	4.71	4.36	-7.4%
KIRC	7.57	6.14	-18.9%
LGG	12.09	5.48	-54.7%
LIHC	5.80	4.27	-26.3%
LUAD	4.19	3.47	-17.4%
LUSC	6.26	3.03	-51.5%
MESO	2.26	1.05	-53.4%
OV	3.71	3.63	-2.2%
PAAD	1.79	1.64	-8.3%
SARC	7.05	4.52	-36.0%
SKCM	3.71	2.25	-39.4%
STAD	3.85	2.09	-45.8%
UCS	2.11	2.30	8.7%
**Average**	**5.05**	**3.47**	**-28.8%**

Cancer Type	Median Survival (yrs)		% Change
	Low EDB-FN	High EDB-FN	
BLCA	5.40	1.82	-66.3%
BRCA	11.69	10.05	-14.0%
ESCA	3.46	1.64	-52.5%
GBM	1.24	0.98	-21.4%
HNSC	4.50	4.79	6.5%
LGG	9.78	5.25	-46.4%
LIHC	4.91	4.27	-12.9%
LUAD	4.15	3.31	-20.3%
LUSC	5.35	3.16	-40.9%
MESO	2.36	1.17	-50.5%
OV	3.71	3.37	-9.3%
PAAD	1.81	1.62	-10.3%
SARC	7.05	5.00	-29.1%
SKCM	3.71	2.25	-39.4%
STAD	3.85	1.95	-49.4%
UCS	1.64	2.30	40.4%
**Average**	**4.66**	**3.31**	**-26.0%**

Supplementary Table S6. Median survival times of patients based on high and low expression of oncofetal fibronectins. Cancer types were excluded from this analysis if the Kaplan-Meier survival curves for either the low or high expression group failed to reach 50% survival.
